# *Bifidobacterium adolescentis* SBT2786-induced early-night sleep requires CCHamide-1 signaling in *Drosophila* ellipsoid body ring neuron circuits

**DOI:** 10.1016/j.isci.2026.117683

**Published:** 2026-09-29

**Authors:** Hiroki Murakami, Shunjiro Kobayashi

**Affiliations:** 1 Milk Science Research Institute, MEGMILK SNOW BRAND Co., Ltd., 1-1-2 Minamidai, Kawagoe, Saitama 350-1165, Japan

**Keywords:** gut-brain axis, *Dosophila melanogaster*, CCHa1-CCHa1R signaling, ellipsoid body neurons, sleep regulation

## Abstract

We previously identified the sleep-promoting bacterium *Bifidobacterium adolescentis* SBT2786; however, its molecular mechanism remains unknown. In *Drosophila melanogaster*, tissue-/region-specific RNA interference (RNAi) targeted the gut-brain neuropeptide CCHamide-1 (CCHa1) and the CCHa1 receptor (CCHa1R). The increase in SBT2786-induced early-night sleep was abolished by systemic CCHa1 and pan-neuronal CCHa1R knockdown. This phenotype persisted after enteroendocrine cell-specific CCHa1 knockdown, suggesting that it is not fully explained by gut enteroendocrine cell-derived CCHa1 alone. Region-specific RNAi revealed that CCHa1R is required in the ellipsoid body (EB), not the fan-shaped body and tested dopaminergic populations. Ring-driver mapping implicated EB ring neuron circuits, including R3-enriched populations, in the SBT2786 effect: CCHa1R knockdown with the 189 years driver abolished the effect, whereas knockdown with drivers labeling R2/R4m populations did not. These findings show that SBT2786-induced early-night sleep requires CCHa1-CCHa1R signaling and EB ring neuron circuits, and is not fully explained by enteroendocrine cell-derived CCHa1.

## Highlights

• SBT2786-induced early-night sleep requires CCHa1-CCHa1R signaling in *Drosophila*
• Enteroendocrine cell-specific CCHa1 knockdown does not abolish the SBT2786 effect
• CCHa1R in ellipsoid body ring neuron circuits is required for sleep promotion
• Ring neuron mapping implicates R3-enriched, but not tested R2/R4m, circuits

## Graphical abstract

**Figure:**
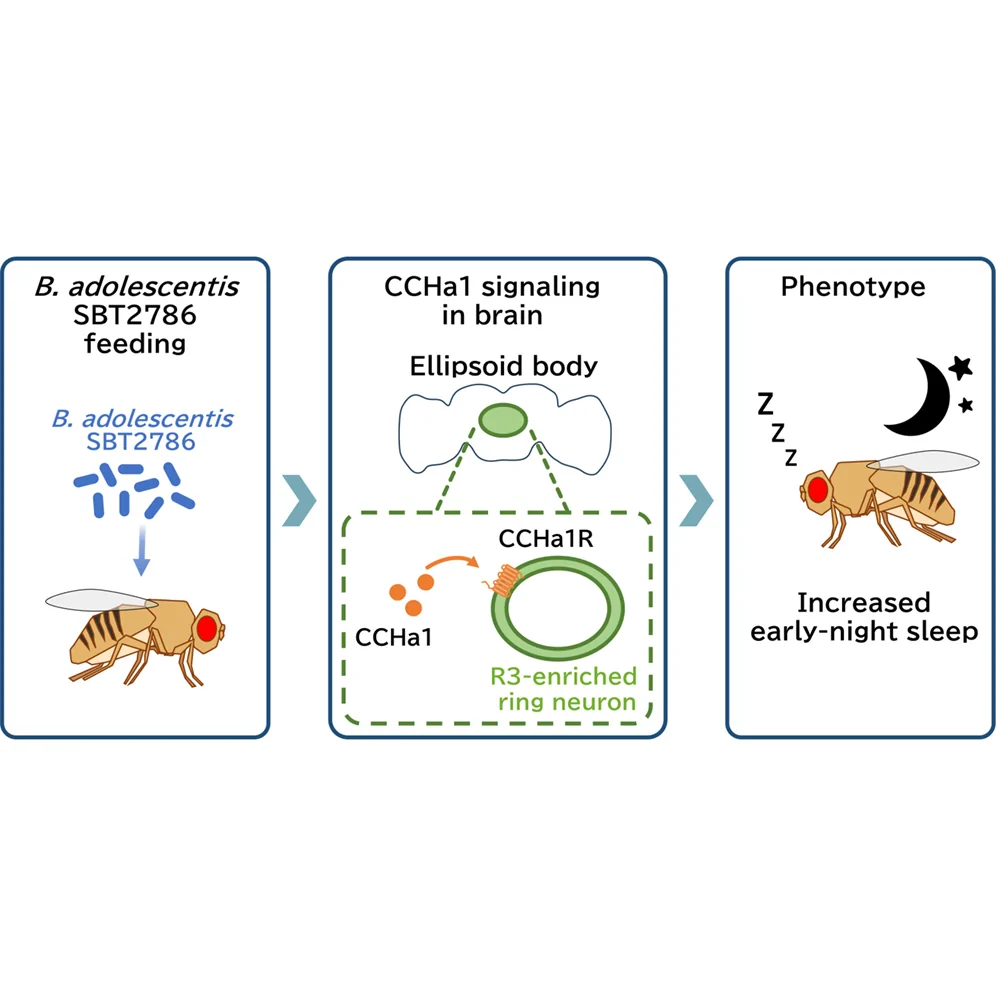

## Teaser

Neuroscience; Microbiome

## Introduction

Sleep disorders are a major public health challenge with wide-ranging effects on individuals and society. Inadequate nocturnal sleep is associated with excessive daytime sleepiness, impaired attention, and decision-making, reduced productivity, and increased risks of obesity, type 2 diabetes, hypertension, and cardiovascular events.^1^^,^^2^ Sleep loss may compromise immune function and amplify inflammatory responses, thereby affecting the course and severity of infectious and chronic diseases.^3^ Beyond somatic health, sleep disturbances are strongly associated with psychiatric conditions, such as depression and anxiety.^4^ At the population level, the high prevalence of sleep disorders imposes a significant economic burden through reduced labor productivity, increased traffic and occupational accidents, and higher healthcare and social security expenditures.^5^ These burdens highlight the urgent need for effective, safe, and scalable interventions to enhance sleep quality without the drawbacks of long-term pharmacotherapy. Non-pharmacological approaches—behavioral strategies, lifestyle modifications, and nutritional interventions—offer specific promise as accessible solutions for public health.

Recently, the gut microbiota has emerged as a primary modulator of host physiology, including neural and endocrine functions, through diverse metabolites, such as short-chain fatty acids and other bioactive compounds, thereby forming the gut-brain signaling.^6^^,^^7^^,^^8^ Converging evidence indicates that the composition and metabolic activity of gut microbes can affect circadian rhythms and sleep phenotypes.^9^^,^^10^^,^^11^ Despite rapid progress, the molecular mechanisms by which microbial signals are transmitted to the brain and their neuronal targets remain unclear.

The fruit fly (*Drosophila melanogaster*) is a powerful genetic model organism for studying sleep biology and gut-brain signaling. Foundational work on flies has identified clock genes through behavioral paradigms,^12^ and sleep-like states were first described in 2000.^13^^,^^14^ Fly sleep shares core organizational principles with mammalian sleep, including circadian and homeostatic regulation,^15^ characteristic transcriptional signatures,^16^ pharmacological responsiveness,^17^ modulation by sex and age,^18^ and neuroendocrine control.^19^ Flies enable cell type-specific manipulation^20^ and temporally controlled gene knockdown.^21^ Combined with sleep measurements, this genetic precision makes flies an effective model for elucidating the molecular basis of microbiota-dependent sleep regulation and mechanistic associations between gut peptides and brain receptors.

We previously identified a specific strain, *Bifidobacterium adolescentis (B. adolescentis*) SBT2786, that increases sleep duration in flies.^22^ Notably, in a human clinical study, SBT2786 consumption enhanced nocturnal sleep duration and subjective satisfaction upon awakening,^23^ supporting the translational relevance from flies to humans. Because the intestine is the primary site where lactic acid bacteria and bifidobacteria interact with the host, enteroendocrine cells (EECs)—that sense luminal cues and secrete peptide hormones into the hemolymph/circulation—are plausible mediators of microbe-elicited brain effects.^24^ These gut-derived peptides can modulate the central circuits governing sleep-wake states and circadian timing. However, direct mechanistic associations connecting specific microbial stimuli to defined peptide-receptor pathways in the brain remain unclear.^25^^,^^26^^,^^27^^,^^28^

Among the candidate mediators, CCHa1 is uniquely positioned to connect intestinal nutrient sensing to arousal and circadian rhythms. Peripherally, EECs release CCHa1 in response to dietary amino acids and signal a restricted set of dopaminergic neurons to modulate arousability.^29^ Centrally, CCHa1 couples anterior dorsal neuron 1 (DN1a) to small ventral lateral clock neurons (s-LNv), shaping the morning activity and daytime siesta timing.^28^ Based on these properties, we hypothesized that CCHa1, acting through its receptor (CCHa1R), can mediate the sleep-promoting effect of SBT2786 and sought to determine whether the CCHa1 source—gut-derived or neuronal—drives this phenotype.

Accumulating evidence indicates that microbial stimuli activate enteroendocrine programs and reshape host physiology and behavior. In fly studies, short-chain fatty acid (SCFA) acetate, a commensal bacteria-derived metabolite, activates the EEC immune deficiency pathway through peptidoglycan recognition protein-LC, thereby increasing tachykinin transcription and affecting lipid metabolism and insulin signaling.^30^ Likewise, pathogen exposure reprograms EEC secretory capacity through the pro-secretory factor dimm, altering allatostatin A levels and antimicrobial responses.^31^ These fly findings parallel mammalian mechanisms in which SCFAs, secondary bile acids, and indoles stimulate L-cell hormone release (glucagon-like peptide-1 and peptide YY) and facilitate L-cell differentiation, establishing a general principle that microbiota-derived metabolites can upregulate enteroendocrine hormone output.^32^^,^^33^ Direct evidence that a specific bacterial strain upregulates CCHa1 has not yet been reported. However, the nutrient-sensing role of CCHa1 and established microbe-EEC hormone pathways provide a rationale to assess whether SBT2786 facilitates sleep by increasing CCHa1 production from EECs.

Based on this hypothesis, our objective was to determine how SBT2786 regulates sleep through CCHa1/CCHa1R signaling. First, we determined whether the peptide is produced in gut EECs or in central nervous system neurons. Second, we examined whether the receptor CCHa1R is required in the brain, particularly within the sleep-regulating central complex. We used RNA interference (RNAi) knockdown and tissue-/region-specific drivers to assess whether disrupting peptide-receptor pairs attenuated the hypnotic effects of SBT2786. We aimed to identify the peptide hormone pathway mediating SBT2786-induced sleep and identify whether gut-derived or central peptides—and their receptors—constitute the crucial axis.

## Results

### GeneSwitch systemic knockdown indicates that CCHa1/CCHa1R signaling is required for early-night sleep promotion by SBT2786

To assess whether CCHa1/CCHa1R signaling was necessary for the sleep-promoting effect of SBT2786, we used RU486-inducible GeneSwitch drivers^21^ to confine the RNAi knockdown to the recording period. Systemic CCHa1 knockdown was induced using tub5-GS Gal4 > (upstream activating sequence) UAS-CCHa1 RNAi and toggled by RU486 (RU−, knockdown OFF; RU+, knockdown ON). Flies were assigned to four groups based on the RU−/RU+ and SBT2786/vehicle conditions. Because our previous studies indicated that SBT2786 exerts its effects on the third night,^22^^,^^34^ we analyzed day-3 sleep architecture (Figures 1A and 1B), total nighttime sleep (zeitgeber time [ZT] 12–24; Figure 1C), and early-night sleep (ZT12–15; Figure 1D). No significant knockdown × treatment interaction was observed during the total nighttime sleep (Figure 1C), whereas SBT2786 increased sleep in RU− controls, and this increase persisted in RU+ knockdown. In contrast, for early-night sleep (ZT12–15), the knockdown × treatment interaction was significant. SBT2786 increased sleep in RU− controls, whereas this effect was abolished in RU+ knockdown (Figure 1D).

**Figure 1:**
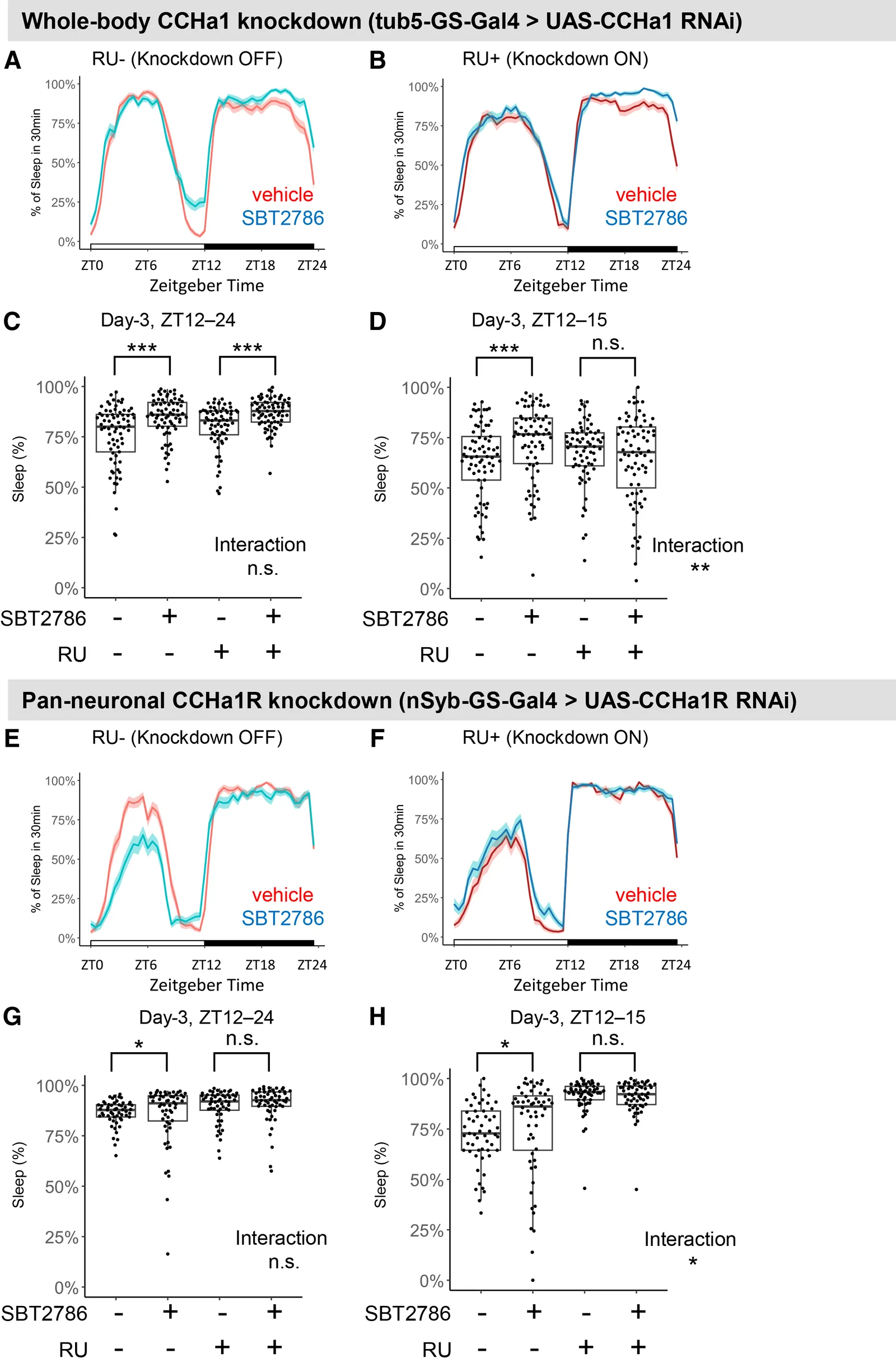
GeneSwitch systemic knockdown of CCHa1/CCHa1R abolishes early-night sleep promotion by SBT2786 (A and B) Day 3 sleep profiles of tub5-GS-Gal4 > UAS-CCHa1 RNAi flies fed vehicle or SBT2786 under RU- (A; GeneSwitch OFF) or RU+ (B; GeneSwitch ON) conditions. (C and D) Total nighttime sleep (ZT12–24) (C) and early-night sleep (ZT12–15) (D) on day 3 for tub5-GS-Gal4 > UAS-CCHa1 RNAi flies. (E and F) Day 3 sleep profiles of nSyb-GS-Gal4 > UAS-CCHa1R RNAi flies fed vehicle or SBT2786 under RU− (E) or RU+ (F) conditions. (G and H) Total nighttime sleep (ZT12–24) (G) and early-night sleep (ZT12–15) (H) on day 3 for nSyb-GS-Gal4 > UAS-CCHa1R RNAi flies. Time-course traces show mean ± SEM (30 min bins). Boxplots show the median (center line), 25th-75th percentiles (box), and whiskers extending to the most extreme data point within 1.5× IQR from the hinge; points indicate individual flies. Thus, the time-course images and summary images represent the same underlying datasets visualized in different formats. Knockdown × treatment interactions were evaluated using the Scheirer-Ray-Hare tests, followed by Wilcoxon-Mann-Whitney post hoc tests. ∗*p* < 0.05, ∗∗*p* < 0.01, ∗∗∗*p* < 0.001; n.s., not significant. Exact statistics for interaction terms, post hoc comparisons, and sample sizes are provided in Table S1.

Because CCHa1 binds CCHa1R expressed throughout the brain,^28^ we assessed whether neuronal CCHa1R mediates this effect by inducing pan-neuronal knockdown (nSyb-GS Gal4 > UAS-CCHa1R RNAi) under a similar RU486 paradigm (Figures 1E and 1F). For total nighttime sleep (ZT12–24), the knockdown × treatment interaction was not significant; however, SBT2786 did not increase sleep under RU+ conditions (Figure 1G). In contrast, the interaction was significant for early-night sleep (ZT12–15), and this effect was abolished by RU+ knockdown (Figure 1H).

Thus, under our experimental conditions, a significant knockdown × treatment interaction was detected in the early-night window (ZT12–15), but not in the whole-night window (ZT12–24). Accordingly, subsequent analyses were focused on ZT12–15.

### SBT2786 promotes sleep independently of gut CCHa1 but requires neuronal CCHa1

Dietary amino acids can be sensed in the gut, triggering CCHa1 release from EECs.^29^ To assess whether gut-derived CCHa1 mediates the sleep-promoting effect of SBT2786, we knocked down CCHa1 in EECs (pros-Gal4 > UAS-CCHa1 RNAi). No significant interaction effect was observed between gut knockdown and SBT2786 treatment, and SBT2786 increased sleep regardless of gut CCHa1 knockdown (Figures 2A–2C). We assessed whether neuronal CCHa1 is required by inducing pan-neuronal knockdown (nSyb-GS Gal4 > UAS-CCHa1 RNAi) under the RU486 GeneSwitch paradigm. In this condition, a significant interaction was detected—SBT2786 increased sleep under RU− conditions but was abolished in RU+ conditions, with sleep reduced relative to the vehicle (Figures 2D–2F).

**Figure 2:**
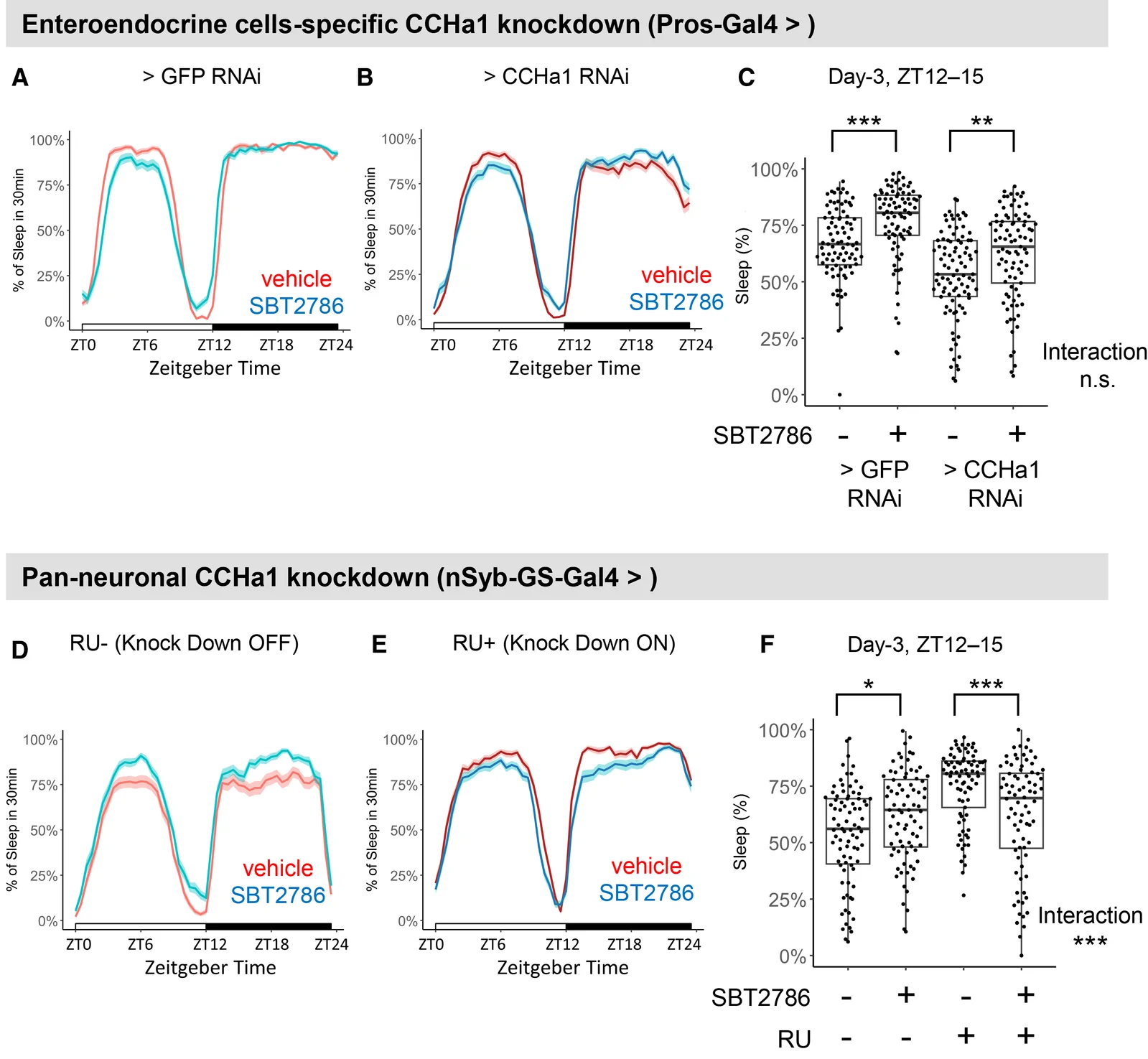
SBT2786 promotes sleep independently of gut CCHa1 but requires neuronal CCHa1 (A and B) Day 3 sleep profile of Pros-Gal4 > UAS-GFP RNAi (A) or Pros-Gal4 > UAS-CCHa1 RNAi (B) flies-fed vehicle or SBT2786. UAS-GFP RNAi lines served as controls in all images. (C) Quantification of day 3 sleep for Pros-Gal4 > UAS-GFP RNAi flies and Pros-Gal4 > UAS-CCHa1 RNAi flies under vehicle/SBT2786 conditions. (D and E) Day 3 sleep profiles of nSyb-GS-Gal4 > UAS-CCHa1 RNAi flies-fed vehicle or SBT2786 under RU− (D; GeneSwitch off) or RU+ (E; GeneSwitch on) conditions. (F) Quantification of day 3 sleep for nSyb-GS-Gal4 > UAS-CCHa1 RNAi flies under RU−/RU+ and vehicle/SBT2786 conditions. Time-course traces show mean ± SEM (30 min bins). Boxplots show the median (center line), 25th–75th percentiles (box), and whiskers extending to the most extreme data point within 1.5× IQR from the hinge; points indicate individual flies. Thus, the time-course images and summary images represent the same underlying datasets visualized in different formats. Knockdown × treatment interactions were evaluated using the Scheirer-Ray-Hare tests, followed by Wilcoxon-Mann-Whitney post hoc tests. ∗*p* < 0.05, ∗∗*p* < 0.01, ∗∗∗*p* < 0.001; n.s., not significant. Exact statistics for interaction terms, post hoc comparisons, and sample sizes are provided in Table S2.

### Brain region-specific knockdowns reveal that EB CCHa1Rs are required

To determine the relevant brain circuitry, we performed region-specific CCHa1R knockdown using constitutive Gal4 drivers, with UAS-GFP RNAi as a control, and quantified day 3 early-night sleep (ZT12–15). In the EB driver R56H10-Gal4,^35^ the knockdown × treatment interaction was significant—SBT2786 increased sleep in the UAS-GFP RNAi controls, whereas it was abolished in UAS-CCHa1R RNAi flies (Figures 3A–3C). Representative confocal immunostaining images of R56H10-Gal4 > UAS-mCD8GFP brains are illustrated in Figure 3D to demonstrate the expression pattern of this driver. In contrast, the interaction of dopaminergic drivers was not significant in TH-Gal4 and protocerebral anterior medial (PAM)-Gal4 for all dopamine neurons and protocerebral anterior medial cluster,^29^^,^^36^ and SBT2786 increased sleep irrespective of knockdown (Figures 3E and 3F). Similarly, in R23E10-Gal4, the interaction of the fan-shaped body (FB) driver was not significant,^37^ and SBT2786 increased sleep regardless of knockdown (Figure 3G).

**Figure 3:**
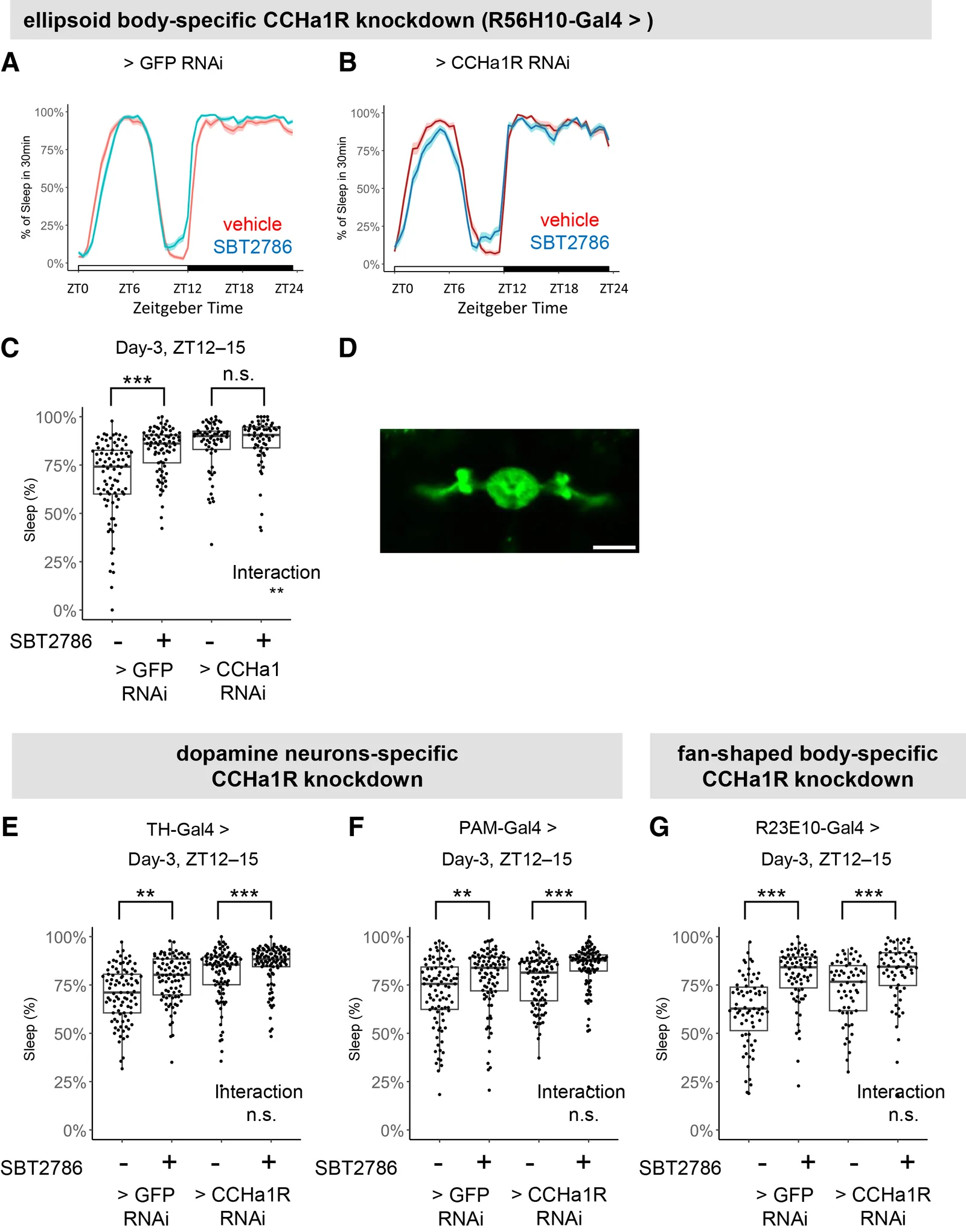
Brain region-specific knockdowns reveal that ellipsoid body CCHa1Rs are required for early-night sleep promotion by SBT2786 (A and B) Day 3 sleep patterns for R56H10-Gal4 > UAS-GFP RNAi (A) or R56H10-Gal4 > UAS-CCHa1R RNAi (B) flies under vehicle or SBT2786. UAS-GFP RNAi lines served as controls in all images. (C) Quantification of day 3 sleep for R56H10-Gal4 flies expressing UAS-GFP RNAi (control) or UAS-CCHa1R RNAi. (D) Representative confocal immunostaining of adult brains from R56H10-Gal4 > UAS-mCD8GFP, illustrating the ellipsoid body labeling pattern of this driver. Scale bars, 50 μm. (E) Early-night sleep (ZT12–15) on day 3 in TH-Gal4 (all dopaminergic neurons) flies expressing UAS-GFP RNAi (control) or UAS-CCHa1R RNAi and fed vehicle or SBT2786. Same analysis for PAM-Gal4 (F) and R23E10-Gal4 (G). Time-course traces show mean ± SEM (30 min bins). Boxplots show the median (center line), 25th–75th percentiles (box), and whiskers extending to the most extreme data point within 1.5× IQR from the hinge; points indicate individual flies. Thus, the time-course panels and summary images represent the same underlying datasets visualized in different formats. Knockdown × treatment interactions were evaluated using the Scheirer-Ray-Hare tests, followed by Wilcoxon-Mann-Whitney post hoc tests. ∗∗*p* < 0.01, ∗∗∗*p* < 0.001; n.s., not significant. Exact statistics for interaction terms, post hoc comparisons, and sample sizes are provided in Table S3.

### Ring-specific mapping implicates R3-enriched EB circuits in the SBT2786 effect

The EB comprises multiple ring neuron classes with distinct functions.^38^^,^^39^^,^^40^ To identify the circuit node required for SBT2786, we knocked down CCHa1R with ring-specific Gal4 drivers and quantified day 3 early-night sleep (ZT12–15), using UAS-GFP RNAi as the control. The knockdown × treatment interaction was significant in the R3-enriched EB ring neuron populations 189 years-Gal4,^41^ and SBT2786 increased early-night sleep in UAS-GFP RNAi controls, while the effect was abolished by UAS-CCHa1R RNAi (Figures 4A–4C). Representative confocal immunostaining images of 189 years-Gal4 > UAS-mCD8GFP brains demonstrate the R3 neuron-labeling pattern (Figure 4D). In contrast, drivers labeling R2/R4m (EB1-Gal4, C819-Gal4)^42^ showed no interaction, with SBT2786 increasing sleep, irrespective of CCHa1R RNAi (Figures 4E and 4F). Similarly, the R2 labeling driver R69F08-Gal4^39^ demonstrated no knockdown × treatment interaction, and SBT2786 significantly increased early-night sleep under both conditions (Figure 4G).

**Figure 4:**
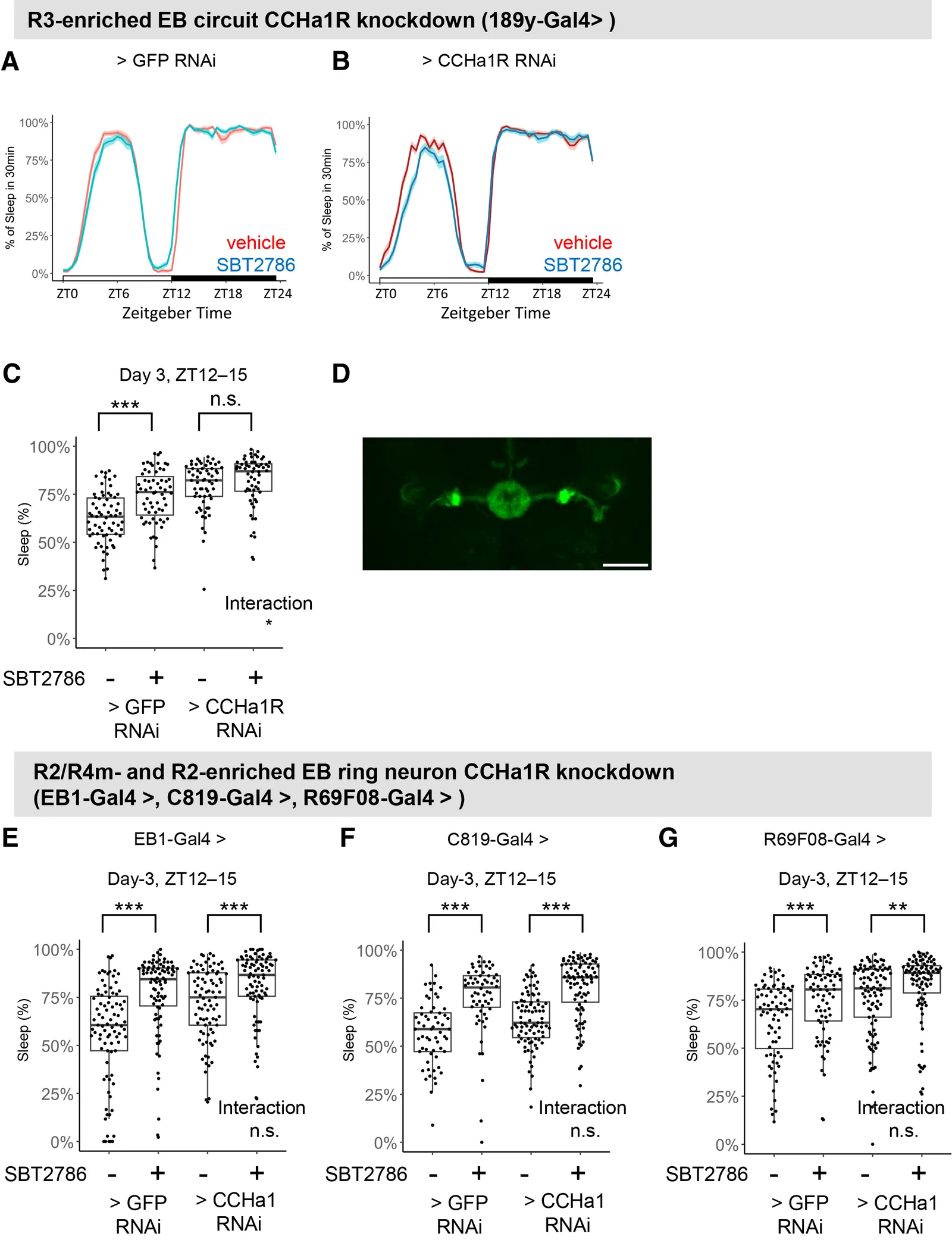
Ring-specific CCHa1R knockdowns implicate EB ring neuron circuits in SBT2786-induced early-night sleep (A and B) Day 3 sleep patterns for 189 years-Gal4 > UAS-GFP RNAi (A) and 189 years-Gal4 > UAS-CCHa1R RNAi (B) flies under vehicle or SBT2786. UAS-GFP RNAi lines served as controls in all images. (C) 189 years-Gal4 (R3-marking driver) with UAS-GFP RNAi control and UAS-CCHa1R RNAi. (D) Representative confocal immunostaining of adult brains from 189 years-Gal4 > UAS-mCD8GFP, illustrating the R3 labeling pattern of this driver. Scale bars, 50 μm. (E and G) R2/R4m drivers: EB1-Gal4 (E), C819-Gal4 (F), and R69F08-Gal4 (R2; G). Time-course traces show mean ± SEM (30 min bins). Boxplots show the median (center line), 25th–75th percentiles (box), and whiskers extending to the most extreme data point within 1.5× IQR from the hinge; points indicate individual flies. Thus, the time-course panels and summary panels represent the same underlying datasets visualized in different formats. Knockdown × treatment interactions were evaluated using the Scheirer-Ray-Hare tests, followed by Wilcoxon-Mann-Whitney post hoc tests. ∗*p* < 0.05, ∗∗*p* < 0.01, ∗∗∗*p* < 0.001; n.s., not significant. Exact statistics for interaction terms, post hoc comparisons, and sample sizes are provided in Table S4.

## Discussion

Probiotic consumption-associated gut microbiota can affect sleep behavior through host physiology. However, the molecular mechanisms and central neural circuits mediating these alterations remain unclear. In this study, we assessed the mode of action of SBT2786 using the tissue-/region-specific genetic knockdowns. Our data indicate that the SBT2786-induced increase in early-night sleep (ZT12–15) requires CCHa1-CCHa1R signaling. Specifically, the early-night sleep increase was abolished by systemic CCHa1 and pan-neuronal CCHa1R knockdown (Figure 1), whereas it was preserved under gut EEC-specific CCHa1 knockdown (Figure 2). These results indicate that under our experimental conditions, EEC-derived CCHa1 is not required for the sleep-promoting effect of SBT2786, whereas pan-neuronal CCHa1 knockdown abolished it. Region-specific knockdown demonstrated that CCHa1R in the EB was required for the SBT2786 effect, whereas its knockdown in tyrosine hydroxylase (TH)/PAM dopaminergic neurons and FB did not abolish this effect (Figure 3). Additionally, ring neuron-specific mapping implicated EB ring neuron circuits, including R3-enriched populations, as the necessary site—CCHa1R knockdown with the 189 years driver abolished the SBT2786-induced sleep increase, whereas knockdown in R2/R4m did not (Figure 4). These findings indicate that, under our experimental conditions, the SBT2786-induced increase in early-night (ZT12–15) sleep requires CCHa1/CCHa1R signaling and CCHa1R function in EB ring neuron circuits, including R3-enriched populations.

In this study, the sleep profiles were consistent with those of previous studies.^22^^,^^34^ Under our experimental conditions, the CCHa1/CCHa1R-dependent component of the SBT2786 phenotype was more clearly resolved in the early-night window (ZT12–15) than across the entire night (ZT12–24). Accordingly, we primarily focused our mechanistic analyses on this interval. Previous reports on CCHa1 mutants have emphasized altered morning activity patterns, including shortened inter-bout intervals, earlier termination of morning activity, and longer daytime siestas, whereas nighttime sleep has received less attention. In the systemic CCHa1 knockdown condition, SBT2786-associated increases in daytime (ZT0–12) and early-morning (ZT0–3) sleep were preserved under both RU− and RU+ conditions (Figure S1), suggesting that these intervals differ from the more clearly resolved early-night CCHa1-dependent component.

To further examine the nighttime structure of the phenotype, we re-analyzed sleep across additional 3 h bins during the dark phase. In this analysis, SBT2786-associated sleep increases were not restricted to ZT12–15, as increases were also observed in later nighttime bins, whereas no interval showed an apparent reduction in sleep under our experimental conditions (Figure S1). Thus, the present data do not support the interpretation that the early-night increase was simply offset by shortened late-night sleep. However, the knockdown × treatment interaction was significant during ZT12–15, but not in the later nighttime intervals. These results suggest that, although SBT2786-associated sleep increases extend beyond the early-night window, the CCHa1-dependent component of the phenotype was most clearly resolved at night onset.

Additional analysis of sleep during ZT9–12 suggested that SBT2786-fed flies may already exhibit elevated sleep before lights off (Figure S1). However, because the interaction between diet and CCHa1 knockdown was not significant in this interval, the contribution of CCHa1 to these pre-lights off effect remains unclear and should be interpreted cautiously. Thus, while a higher sleep level before ZT12 may contribute in part to the apparent early-night phenotype, the present data do not support a strong mechanistic conclusion for this interval.

Because CCHa1 has also been implicated in regulating daytime siesta, we additionally analyzed daytime and early-morning sleep in the pan-neuronal CCHa1 knockdown condition (nSyb-GS-Gal4 > UAS-CCHa1 RNAi). In these analyses, a knockdown × treatment interaction was detected in ZT0–3, although the SBT2786 effect did not reach significance within each RU condition, and a significant interaction was also observed in ZT0–12, where SBT2786 increased sleep under RU− conditions but decreased sleep under RU+ conditions (Figure S1). These findings suggest that neuronal CCHa1 may also contribute to the daytime/early-morning component of the SBT2786 phenotype. However, the direction and clarity of the effects differed across time windows, indicating that the role of neuronal CCHa1 during the day is more complex than a simple uniform sleep-promoting effect. Taken together, the present data indicate that neuronal CCHa1 contributes to SBT2786-associated sleep regulation in a time-of-day-dependent manner, with the most clearly interpretable CCHa1-dependent component detected during ZT12–15.

CCHa1 is expressed in various tissues. In the gut, CCHa1 is secreted by EECs in response to dietary amino acid levels. EEC-secreted CCHa1 has been implicated in modulating arousal thresholds and feeding behavior.^29^^,^^43^ In the brain, CCHa1 serves as a coupling signal in the circadian clock network by relaying signals from DN1a to s-LNv, thereby regulating morning activity and daytime siesta timing.^28^ To determine the source of CCHa1 responsible for the SBT2786 effect, we selectively knocked down CCHa1 in the gut and nervous system. EEC-specific knockdown did not abolish the SBT2786-induced sleep increase, whereas nSyb-GS-driven knockdown eliminated it. These findings indicate that the SBT2786-induced sleep phenotype is not fully explained by gut EEC-derived CCHa1 alone. However, because the nSyb driver may also label EECs,^44^ the present data do not formally establish an exclusively neuronal source of CCHa1, and some contribution of EEC-related mechanisms cannot be completely excluded. Although EEC-CCHa1 is dispensable, SBT2786 may still engage in gut-originating processes that indirectly affect central sleep regulatory pathways. Potential mediators include microbial metabolites,^45^^,^^46^ host immune or epithelial signaling,^47^ and neuronal routes innervating the gut that are functionally analogous to the vertebrate vagal afferent pathways.^48^ Further studies are required to clarify whether and how these upstream mechanisms contribute to the SBT2786 phenotype. At present, however, we do not know which of these upstream factors are induced by SBT2786 or how such signals ultimately reach EB ring neuron circuits.

Using brain region-specific knockdowns, we identified that the sleep-promoting effect of SBT2786 depends on CCHa1R in the EB, a component of the central complex. The central complex has been implicated in wake/sleep regulation, motor control, and integration of orientation and internal state, and its functions have been compared with those of the basal ganglia in mammals.^49^^,^^50^ In contrast, knockdown of the PAM dopaminergic system or FB did not eliminate sleep promotion (Figure 3). Although PAM/TH dopaminergic neurons contribute to nutritional state-dependent arousal thresholds^29^ and the dorsal FB has been proposed as a sleep homeostasis center, neither mediated the increase in sleep under our conditions. The EB comprises multiple cell populations, including anatomically distinct classes of ring neurons categorized into subclasses based on their morphology and connectivity.^38^^,^^51^ Ring neurons typically extend dendrites into the bulb, located lateral to the EB, and project axons as concentric rings within the EB. R2/R4m activation can increase sleep,^39^ whereas R3d activation facilitates wakefulness and R3m activation induces sleep. Both R3m and R3d release gamma-aminobutyric acid, and R3m-mediated inhibition of R3d has been proposed as a local motif.^40^^,^^52^ In our driver-based mapping, the 189 years line, which labels R3-enriched populations, showed loss of the SBT2786 effect upon CCHa1R knockdown, whereas EB1, C819, and R69F08 did not. Thus, our data support the involvement of EB ring neuron circuits, including R3-enriched populations, but do not by themselves establish that a narrowly defined R3 subtype is the unique critical locus. Therefore, CCHa1R signaling in EB ring neuron circuits, including R3-enriched populations, may represent one downstream node through which upstream SBT2786-dependent signals are translated into increased early-night sleep.

An important consideration is the potential contribution of nutritional factors, because the experimental diet consisted of sucrose/agar with or without bacterial biomass supplementation. Thus, under the present dietary conditions, nutrient-related effects should be carefully considered and cannot be completely excluded. However, our previous study included a direct strain-level comparison between SBT2786 and another *B. adolescentis* strain, BA003.^22^ Although the initial screening was standardized by culture volume, the subsequent SBT2786-BA003 comparison used washed and lyophilized bacterial preparations incorporated into the fly food at the same concentration of approximately 1% (w/v). Under these conditions, SBT2786 increased nighttime sleep, whereas BA003 did not produce comparable changes in the sleep parameters examined. Thus, the differential phenotype cannot readily be explained by differences in nominal bacterial dry-biomass concentration alone. Nevertheless, equal nominal dry-biomass concentration does not ensure equivalence in protein content, amino acid composition, caloric value, micronutrient composition, or digestibility. Therefore, strain-dependent nutritional differences may have contributed to the differential phenotype. However, the available evidence does not demonstrate that generic nutritional rescue is sufficient to explain the SBT2786-associated sleep phenotype. The relevant strain-specific properties of the SBT2786 preparation may include differences in nutritional composition, other bacterial components, or a combination of both. Their biochemical identity and relative contributions of these factors remain to be determined. Importantly, this uncertainty regarding the upstream SBT2786-derived input does not affect the interpretation of the principal genetic findings of the present study. Our findings indicate that CCHa1-CCHa1R signaling and CCHa1R function in EB ring neuron circuits are required for the SBT2786-associated early-night sleep phenotype under the dietary conditions used here.

### Limitations of the study

This study has several limitations. First, although we demonstrated that CCHa1/CCHa1R is necessary for the SBT2786-induced increase in sleep, its sufficiency remains unclear. Future experiments should determine whether time-locked activation of R3 neurons at the beginning of the night can recapitulate the sleep increase and whether rescuing CCHa1R in R3 neurons can restore the effect of SBT2786. Second, the source of CCHa1 responsible for the SBT2786 phenotype has not yet been identified. As discussed earlier, the phenotype is not fully explained by gut EEC-derived CCHa1 alone, but the present data do not define the precise CCHa1-producing cell population. The neuronal populations that release CCHa1 and contribute to the SBT2786-induced sleep increase remain unclear. Additionally, it remains unclear whether R3 signaling is mediated by direct connectivity, polysynaptic pathways, or volume transmission. Third, the coverage of region-specific GAL4 lines was limited. Importantly, the ring-driver lines used here are EB-enriched but not single-cell-type specific; therefore, the present data support the involvement of EB ring neuron circuits, including R3-enriched populations, rather than a uniquely defined R3 subtype. Fourth, all sleep analyses in this study were performed in virgin females. This design was chosen to minimize variability arising from mating status and to evaluate sleep-promoting effects under conditions with sufficient assay sensitivity. Because *Drosophila* sleep is sexually dimorphic and males generally show higher baseline sleep levels than females, the dynamic range for detecting further sleep promotion may be more limited in males. Therefore, whether the sleep-promoting effect of SBT2786 is retained in mated females or similarly observed in males remains unclear and should be addressed in future studies. Finally, as discussed earlier, strain-dependent nutritional contributions cannot be completely excluded under the present dietary conditions, and the biochemical identity and relative contributions of the relevant strain-specific components of the SBT2786 preparation remain unresolved.

In summary, the early-night sleep-promoting effect of SBT2786 in flies required a CCHa1-dependent pathway and CCHa1R expressed in the EB. The effect was not abolished by EEC-specific CCHa1 knockdown, suggesting that the phenotype is not fully explained by gut EEC-derived CCHa1 alone. Region- and ring-specific mapping implicated EB ring neuron circuits, including R3-enriched populations, in this phenotype, whereas TH/PAM dopaminergic and FB circuits were not required. Testing subtype-specific sufficiency and necessity in more selective EB neuron populations, performing CCHa1R rescue, identifying upstream mediators, and increasing subtype resolution should advance a more precise circuit- and molecular-level model of how SBT2786 promotes sleep.

## Resource availability

### Lead contact

Further information and requests for resources should be directed to and will be fulfilled by the lead contact Hiroki Murakami (hiroki-murakami@meg-snow.com).

### Materials availability

*B. adolescentis* SBT2786 was deposited at the National Institute of Technology and Evaluation Patent Microorganisms Depositary (Chiba, Japan) and is available from that institution. The accession number is NITE BP-03102.

### Data and code availability

• Data reported in this paper will be shared by the lead contact upon request.
• This paper does not report original code.
• Any additional information required to reanalyze the data reported in this paper is available from the lead contact upon request.

## Acknowledgments

We thank Dr. H. Ishimoto, Bloomington Drosophila Stock Center for providing fly stocks. We are grateful to Masaki Kumai, Mie Kasuga, and Emiko Yamaguchi (MEGMILK SNOW BRAND Business Solution Co., Ltd.) for assistance with fly maintenance.

## Author contributions

H.M. and S.K. designed research; H.M. performed research; H.M. and S.K. analyzed data; H.M. wrote the paper; and S.K. reviewed and edited the paper. All authors read and approved the manuscript.

## Declaration of interests

H.M. and S.K. are employees of MEGMILK SNOW BRAND Co., Ltd. H.M. is an inventor on a granted patent related to the sleep-promoting effects of SBT2786 (Japan, patent no. P6873291). H.M. and S.K. are also inventors on pending patent applications concerning the anti-stress effects of SBT2786 and the SBT2786-induced upregulation of CCHa1 expression (Japan, JP2024-052320, JP2026-048318).

## Declaration of generative AI and AI-assisted technologies in the writing process

During manuscript preparation, we used Smart Generative Chat (model: GPT-5; System Support Inc.) and Microsoft 365 Copilot (Microsoft Corporation) to assist with the organization, text clarity, and English translation. All AI-generated or suggested outputs were reviewed and edited by the authors, who took full responsibility for the final manuscript.

## STAR★Methods

### Key resources table

**Table:** 

REAGENT or RESOURCE	SOURCE	IDENTIFIER
**Antibodies**		

rabbit polyclonal anti-GFP	MEDICAL & BIOLOGICAL LABORATORIES CO., LTD.	Cat# 598, RRID:AB_591819
Goat Anti-Rabbit IgG H&L (Alexa Fluor® 488)	Abcam Limited.	Cat# ab150077, RRID:AB_2630356

**Bacterial and virus strains**		

*Bifidobacterium adolescentis* SBT2786	This paper	RRID: N/A

**Chemicals, peptides, and recombinant proteins**		

RU486 (mifepristone)	Sigma-Aldrich	Cat# M8046
Sucrose	NACALAI TESQUE, INC.	Cat# 30404-45
BD Bacto Agar	BD	Cat# 214010
Normal Goat Serum	Abcam Limited.	Cat# ab7481, RRID:AB_2716553
VECTASHIELD Mounting Medium with DAPI	VECTOR LABORATORIES, INC.	Cat# H-1200-10

**Critical commercial assays**		

RNeasy mini kit	Qiagen	Cat# 74104
ReverTra Ace® qPCR RT Master Mix with gDNA Remover	TOYOBO	Cat# FSQ-301
THUNDERBIRD® Next SYBR™ qPCR Mix	TOYOBO	Cat# QPX-201

**Experimental models: Organisms/strains**		

*D. melanogaster*: Canton-S2202u	Dr. Ishimoto, Nagoya Univ. Japan	RRID: N/A
*D. melanogaster*: tub5-GS-Gal4	Dr. Ishimoto, Nagoya Univ. Japan	RRID: N/A
*D. melanogaster*: UAS-CCHa1-RNAi: y[1] sc[∗] v[1] sev[21]; P{y[+t7.7] v[+t1.8]=TRiP.HMC04879}attP40	Bloomington Drosophila Stock Center	RRID: BDSC_57562
*D. melanogaster*: UAS-CCHa1R-RNAi: y[1] v[1]; P{y[+t7.7] v[+t1.8]=TRiP.HMJ21029}attP40	Bloomington Drosophila Stock Center	RRID: BDSC_51168
*D. melanogaster*: UAS-NPF-RNAi: y[1] v[1]; P{y[+t7.7] v[+t1.8]=TRiP.JF02555}attP2	Bloomington Drosophila Stock Center	RRID: BDSC_27237
*D. melanogaster*: UAS-NPFR-RNAi: y[1] v[1]; P{y[+t7.7] v[+t1.8]=TRiP.JF01959}attP2	Bloomington Drosophila Stock Center	RRID: BDSC_25939
*D. melanogaster*: UAS-GFP-RNAi: y[1] sc[∗] v[1] sev[21]; P{y[+t7.7] v[+t1.8]=VALIUM20-EGFP.RNAi.4}attP40	Bloomington Drosophila Stock Center	RRID: BDSC _41552
*D. melanogaster*: UAS-mCD8:GFP: w[1118]; P{y[+t7.7] w[+mC]=10XUAS-IVS-mCD8:GFP}su(Hw)attP1	Bloomington Drosophila Stock Center	RRID: BDSC _32187
*D. melanogaster*: EB1-Gal4: y[1] w[∗]; betaTub60D[Pin-1]/CyO; P{w[+mW.hs]=GawB}EB1	Bloomington Drosophila Stock Center	RRID: BDSC_44409
*D. melanogaster*: 189years-Gal4: w[∗]; P{w[+mW.hs] = GawB}lilli[189Y]	Bloomington Drosophila Stock Center	RRID: BDSC_30817
*D. melanogaster*: C232-Gal4: w[∗]; P{w[+mW.hs] = GawB}Alp4[c232]	Bloomington Drosophila Stock Center	RRID: BDSC_30828
*D. melanogaster*: C819-Gal4: w[∗]; P{w[+mW.hs]=GawB}c819	Bloomington Drosophila Stock Center	RRID: BDSC_30849
*D. melanogaster*: R69F08-Gal4: w[1118]; P{y[+t7.7] w[+mC]=GMR69F08-GAL4}attP2	Bloomington Drosophila Stock Center	RRID: BDSC_39499
*D. melanogaster*: R56H10-Gal4: w[1118]; P{y[+t7.7] w[+mC]=GMR56H10-GAL4}attP2/TM3, Sb[1]	Bloomington Drosophila Stock Center	RRID: BDSC_61644
*D. melanogaster*: R23E10-Gal4: w[1118]; P{y[+t7.7] w[+mC]=GMR23E10-GAL4}attP2	Bloomington Drosophila Stock Center	RRID: BDSC_ 49032
*D. melanogaster*: nSyb-GS-Gal4: w[1118]; P{y[+t7.7] w[+mC]=nSyb-GS.B}attP2	Bloomington Drosophila Stock Center	RRID: BDSC_80699
*D. melanogaster*: TH-Gal4: w[∗]; P{w[+mC]=ple-GAL4.F}3	Bloomington Drosophila Stock Center	RRID: BDSC_8848
*D. melanogaster*: PAM-Gal4: w[1118]; P{y[+t7.7] w[+mC]=GMR58E02-GAL4}attP2	Bloomington Drosophila Stock Center	RRID: BDSC_41347

**Software and algorithms**		

R Project for Statistical Computing 4.4.0	R Core Team	https://www.r-project.org; RRID: SCR_001905
R Studio 1.4.1106	RStudio PBC	https://www.rstudio.com/; RRID: SCR_000432
Rethomics	Geissmann et al.^53^	https://rethomics.github.io/; RRID: N/A

**Other**		

TriKinetics Drosophila Activity Monitoring System, DAM2	Trikinetics	https://trikinetics.com; RRID: SCR_021798
Olympus FV1000 Confocal Microscope	Olympus	RRID:SCR_020337

### Experimental model and study participant details

#### Fly strains and rearing conditions

All fly strains used in this study were reared on standard cornmeal–yeast food (50 g/L glucose, 45 g/L yeast, 40 g/L corn flour, 8 g/L agar, 4 mL/L propionic acid, and 3 mL/L methyl 4-hydroxybenzoate) at 24 ±1 °C and 60 ± 10% humidity under a 12 h light/12 h dark cycle. Canton-S strain was used as the wild-type control.

We used the Gal4/UAS binary expression system to express RNAi constructs in defined tissues or neuronal populations. For temporally restricted knockdown, GeneSwitch Gal4 drivers were activated by RU486 only during the behavioral recording period. To drive RNAi expression, we used both RU486-inducible GeneSwitch Gal4 drivers and constitutive tissue-/region-specific Gal4 drivers. The inducible drivers were tub5-GS-Gal4 (provided by Dr. Hiroshi Ishimoto, Nagoya University, Japan) and nSyb-GS-Gal4 (BDSC_80699).

Constitutive Gal4 drivers used for tissue- or region-specific knockdown were Pros-Gal4, R56H10-Gal4 (BDSC_61644), TH-Gal4 (BDSC_8848), PAM-Gal4 (BDSC_41347), R23E10-Gal4 (BDSC_49032), 189years-Gal4 (BDSC_30817), EB1-Gal4 (BDSC_44409), C819-Gal4 (BDSC_30849), and R69F08-Gal4 (BDSC_39499). UAS effector lines were UAS-CCHa1-RNAi (BDSC_57562), UAS-CCHa1R-RNAi (BDSC_51168), and UAS-GFP-RNAi (BDSC_41552). UAS-mCD8GFP (BDSC_32187) was used to visualize driver expression patterns. Bloomington lines were obtained from the Bloomington Drosophila Stock Center. Gal4 lines were backcrossed to Canton-S at least six times before use. For all experiments, virgin females were collected under CO_2_ anesthesia and maintained in food vials (15–20 flies per vial).

### Method details

#### Preparation of lactic acid bacteria samples

*B. adolescentis* SBT2786 was obtained from MEGMILK SNOW BRAND Co., Ltd. (Tokyo, Japan) and cultured in Gifu anaerobic medium broth (Shimadzu Diagnostics Corporation, Tokyo, Japan) supplemented with 1% (w/v) glucose at 37 °C for 18 h under anaerobic conditions. Subsequently, the cells were collected by centrifugation (10,000 × *g*, 20 min, 4 °C), washed once with sterilized saline (0.9% NaCl) and once with sterilized ultrapure water, and concentrated by centrifugation. The concentrated bacterial suspension was frozen, lyophilized to obtain a powdered preparation, and stored at -20 °C.

### Quantification and statistical analysis

#### Sleep analysis

Sleep was assessed using the *Drosophila* Activity Monitoring System (TriKinetics, Waltham, MA, USA). Virgin females (3–5 days old) were individually placed in glass tubes (5 × 65 mm) containing either control or test food at one end and sealed with a cotton plug at the other end. The flies were acclimated overnight before recording. Locomotor activity was monitored for 3 days at 24 ± 1 °C under a 12 h light/dark cycle.

The control diet contained 5.0% (w/v) sucrose and 1.0% (w/v) agar. The test diets were supplemented with 1.5% (w/v) SBT2786. For GeneSwitch experiments, RU486 (mifepristone; Sigma-Aldrich, MO, USA) was added to the food at a final concentration of 500 μM, and control food received an equal volume of vehicle (70% ethanol).

Activity data were collected in 1-min bins. Periods of inactivity lasting ≥ 5 min were defined as sleep bouts, as previously described.^13^^,^^14^ Based on our previous studies, day 3 was selected as the primary time point for mechanistic analyses because the sleep-promoting effect of SBT2786 is most consistently observed at this stage under our experimental conditions. To assess whether the phenotype was distributed across the night or preferentially enriched at night onset, we analyzed both total nighttime sleep (ZT12–24) and early-night sleep (ZT12–15). Behavioral parameters were calculated for individual flies using the R package Rethomics.^53^ Dead flies were excluded from the analysis. Total sleep was calculated as the sum of all sleep bout durations within a specified time window. For plotting, sleep within each analysis window was expressed as Sleep (%) (sleep time divided by the window length × 100).

#### Immunostaining of *Drosophila* brains

Fly brains were dissected in phosphate-buffered saline (PBS) (pH 7.4) and fixed in PBS containing 0.05% Triton X-100 (PBST) supplemented with 4% paraformaldehyde for 1 h at 25 °C, followed by two washes in PBST. Subsequently, the samples were blocked for 1 h in an antibody diluent (PBST containing 10% normal goat serum; ab7481; Abcam, Cambridge, UK). Primary antibodies were diluted in the antibody diluent (rabbit polyclonal anti-GFP, 1:500; MEDICAL & BIOLOGICAL LABORATORIES Co., Ltd., Tokyo, Japan). After removing PBST, the brains were incubated with the primary antibody solution at 4 °C overnight with gentle agitation. After two washes in PBST, secondary antibodies were diluted in antibody diluent (Goat Anti-Rabbit IgG H&L; [Alexa Fluor 488], Abcam, ab150077; 1:300). After removing PBST, the brains were incubated with the secondary antibody solution at 4 °C overnight with gentle agitation. The brains were washed twice with PBST, mounted in VECTASHIELD mounting medium, and imaged using an FLUOVIEW FV1000 confocal laser scanning microscope (Olympus, Tokyo, Japan).

#### Statistical analysis

All statistical analyses were performed using R software (version 4.4.0). For sleep data, non-parametric methods were applied. Interactions between knockdown status and SBT2786 treatment were assessed using the Scheirer–Ray–Hare test, a nonparametric alternative to the two-way analysis of variance implemented in the rcompanion package.^54^ This method extends the Kruskal–Wallis test to factorial designs, enabling the assessment of primary effects and interactions without assuming normality. When a significant interaction was detected, straightforward primary effects were assessed using the Wilcoxon–Mann–Whitney tests. Statistical significance was set at *p* < 0.05. Box-and-whisker plots demonstrate the median and interquartile range (distance between the first and third quartiles), with whiskers representing the minimum and maximum values, excluding outliers.

## Supplemental information

Document S1. Figures S1

Table S1. Statistical analyses of sleep in tub5-GS-Gal4> and nSyb-GS-Gal4> flies, related to Figure 1 This table reports the results of Scheirer-Ray-Hare tests and post hoc Wilcoxon-Mann-Whitney tests, including test statistics, *p* values, and sample sizes (*n*), for the indicated genotypes and treatments

Table S2. Statistical analyses of sleep in Pros-Gal4> and nSyb-GS-Gal4> flies, related to Figure 2 This table reports the results of Scheirer-Ray-Hare tests and post hoc Wilcoxon-Mann-Whitney tests, including test statistics, *p* values, and sample sizes (*n*), for the indicated genotypes and treatments

Table S3. Statistical analyses of sleep across distinct neuronal populations, related to Figure 3 This table reports the results of Scheirer-Ray-Hare tests and post hoc Wilcoxon-Mann-Whitney tests, including test statistics, *p* values, and sample sizes (*n*), for the indicated neuronal subtypes and treatments

Table S4. Statistical analyses of sleep across distinct ellipsoid body ring neuron subtypes, related to Figure 4 This table reports the results of Scheirer-Ray-Hare tests and post hoc Wilcoxon-Mann-Whitney tests, including test statistics, *p* values, and sample sizes (*n*), for the indicated ring neuron subtypes and treatments

Table S5. Statistical analyses of sleep in tub5-GS-Gal4> and nSyb-GS-Gal4> flies, related to Figure S1 This table reports the results of Scheirer-Ray-Hare tests and post hoc Wilcoxon-Mann-Whitney tests, including test statistics, *p* values, and sample sizes (*n*), for the sleep analyses of the indicated genotypes and treatments
