# Supplementary material for: Bifidobacterium adolescentis SBT2786-induced early-night sleep requires CCHamide-1 signaling in Drosophila ellipsoid body ring neuron circuits
Source: iScience. 2026 Sep 29;29(10):117683. doi: 10.1016/j.isci.2026.117683 (PMC13654555; doi:10.1016/j.isci.2026.117683)
Supplement: Supplementary file 1 [file mmc1.pdf]

iScience, Volume *29*

Supplemental information

Bifidobacterium adolescentis SBT2786-induced early-night sleep requires CCHamide-1 signaling in *Drosophila* ellipsoid body ring neuron circuits

Hiroki Murakami and Shunjiro Kobayashi

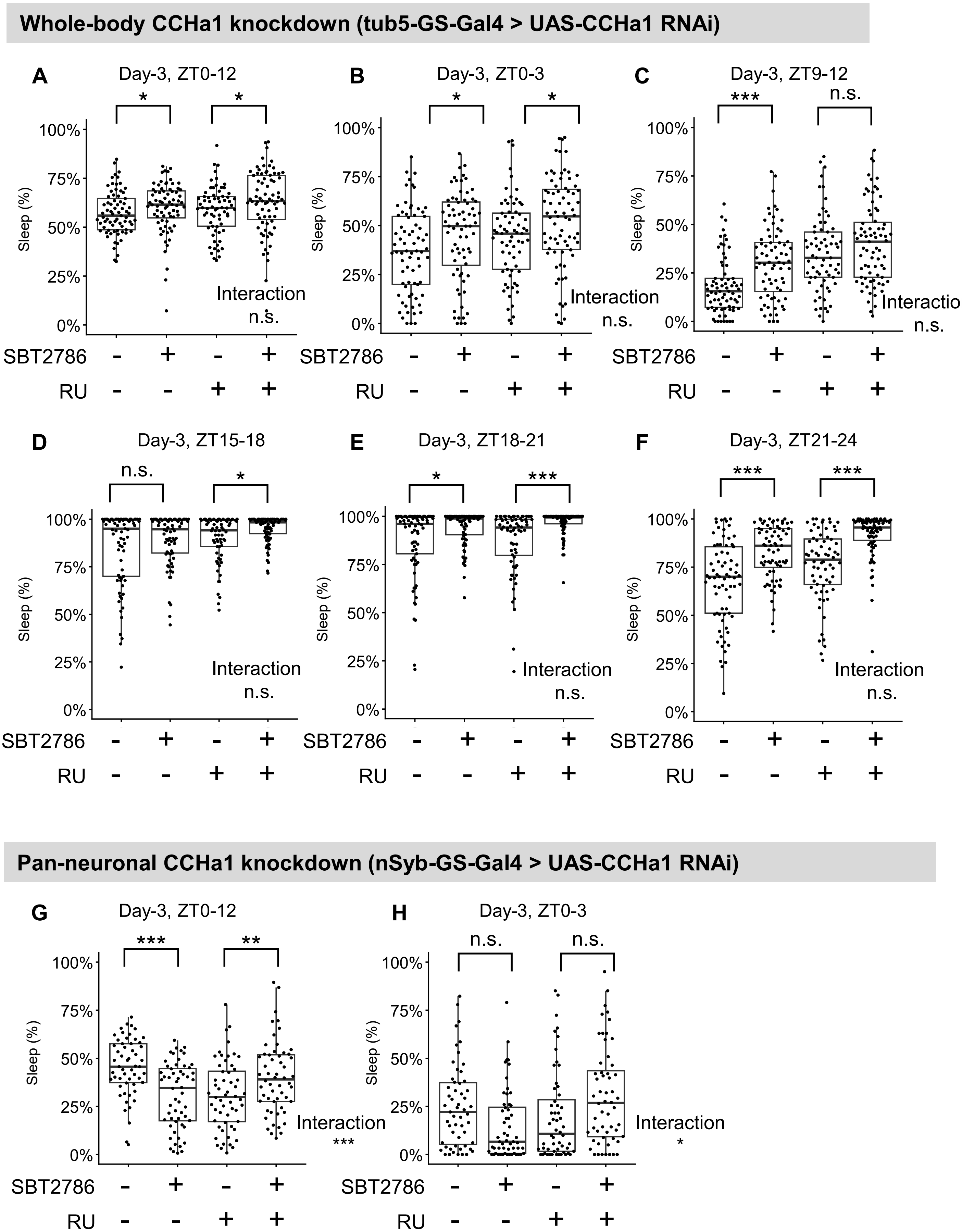

Whole-body CCHa1 knockdown (tub5-GS-Gal4 > UAS-CCHa1 RNAi)
A
Day-3, ZT0-12
*
*
Interaction n.s.
B
Day-3, ZT0-3
*
*
Interaction n.s.
C
Day-3, ZT9-12
***
n.s.
Interaction n.s.
D
Day-3, ZT15-18
n.s.
*
Interaction n.s.
E
Day-3, ZT18-21
*
***
Interaction n.s.
F
Day-3, ZT21-24
***
***
Interaction n.s.
Pan-neuronal CCHa1 knockdown (nSyb-GS-Gal4 > UAS-CCHa1 RNAi)
G
Day-3, ZT0-12
***
**
Interaction ***
H
Day-3, ZT0-3
n.s.
n.s.
Interaction *
Sleep (%)
0%
25%
50%
75%
100%
SBT2786 - + - +
RU - - + +

Supplemental Figure1 SBT2786 increases daytime and early-morning sleep independently of tub5-GS-driven CCHa1 knockdown.

Day-3 sleep profiles and quantification of daytime sleep in tub5-GS-Gal4 > UAS-CCHa1 RNAi flies fed vehicle or SBT2786 under RU− or RU+ conditions. Daytime sleep (ZT0–12) (A), early-morning sleep (ZT0–3) (B), late-day sleep before lights-off (ZT9–12) (C), and nighttime sleep in 3-h bins during ZT15–18 (D), ZT18–21 (E), and ZT21–24 (F) are shown. Daytime sleep (ZT0–12) (G) and early-morning sleep (ZT0–3) (H) were also analyzed in nSyb-GS-Gal4 > UAS-CCHa1 RNAi flies fed vehicle or SBT2786 under RU− or RU+ conditions.

Time-course traces show mean ± SEM (30-min bins). Boxplots show the median (center line), 25th–75th percentiles (box), and whiskers extending to the most extreme data point within 1.5× IQR from the hinge; points indicate individual flies. Thus, the time-course panels and summary panels represent the same underlying datasets visualized in different formats.

Knockdown × treatment interactions were evaluated using the Scheirer–Ray–Hare tests, followed by Wilcoxon–Mann–Whitney post hoc tests. *p<0.05; n.s., not significant.

Exact statistics for interaction terms, post hoc comparisons, and sample sizes are provided in Supplementary Table S5.
